# Solitary juvenile xanthogranuloma of temporal bone: a case report

**DOI:** 10.1186/s12887-022-03150-3

**Published:** 2022-02-12

**Authors:** Shu-ni Wang, Ji Lu

**Affiliations:** The Department of Radiology, The First College of Clinical Medical Science of China Three Gorges University and Yichang Central People’s Hospital, Yichang, 443000 Hubei China

**Keywords:** Juvenile xanthogranuloma, Solitary, Temporal bone, Histiocytosis, Case report

## Abstract

**Background:**

Juvenile xanthogranuloma (JXG) is a kind of non-Langerhans cell histiocytosis, usually with skin lesions as the main manifestation. It rarely occurs in other tissues or organs and even more rarely is it found in the skull. Here, we report a case of xanthogranuloma derived from the temporal bone that was not present in any other parts of the body.

**Case presentation:**

A 15-year-old boy had an unaccountable right migraine for 7 months. A mass with tenderness was located behind his right ear. The mass gradually increased in size, and his headache continued. Eventually, he came to our hospital for treatment. A computed tomography (CT) scan and magnetic resonance imaging (MRI) revealed a cystic solid mass in the right temporo-occipital region with skull destruction. The clinical diagnosis was haemangiopericytoma and skull-derived tumour. Haematological and biochemical results were as follows: alanine aminotransferase (ALT) 7 U/L; aspartate aminotransferase (AST) 12 U/L; basophil percentage (BASO%) 1.2%; normal coagulation. The patient was successfully treated with total surgical resection of the tumour. Postoperative histopathology examination showed xanthogranuloma, and his prognosis after surgery was good.

**Conclusions:**

Because JXG rarely occurs in the skull and lacks typical imaging findings, an accurate diagnosis is difficult. The diagnosis of this disease mainly depends on pathological examination and immunohistochemistry. If feasible, many intracranial lesions can be cured through complete resection.

## Background

Juvenile xanthogranuloma (JXG) is also known as cholesterol granuloma or nevo-xanthoendothelioma. Adamson first reported JXG in 1905 and called it congenital xanthoma multiplex. JXG is not a true neoplasm but reactive proliferation of histiocytes, which may originate from a subgroup of non-Langerhans dendritic cells called dermal dendritic cells [1] and is the most common non-Langerhans cell histiocytosis. The disease usually occurs in infants, children, and adolescents. The pathogenesis and predisposing factors remain unclear. JXG is usually characterized by skin lesions on the head and neck and typically presents as soft yellow papules or nodules of different sizes ranging from a few millimetres to several centimetres. These solitary or multiple cutaneous nodules usually regress spontaneously. Although more than 90% of JXG patients only have skin lesions, sometimes internal organs such as the liver, spleen, lung, kidney, eyes, subcutaneous soft tissue, bones, and central nervous system (CNS) are involved [2]. Skeletal JXG manifestations of systemic cases have also been reported, but isolated bone lesions are rare. We searched PubMed Central to identify peer-reviewed studies of skull JXG and found reports of only 4 cases of isolated skull JXG since 1997 [1, 3–5]. Clinical symptoms of isolated skull JXG vary depending on the location of the mass. For example, mastoid involvement may lead to hearing loss. Regardless, there is always pain and swelling around the mass due to the mass effect. In view of its destruction to bone and to prevent any further damage, such as neurological complications, the usual strategy is tumour resection, after which the prognosis is usually good. However, when resection is incomplete, local recurrence is likely to occur, even though JXG is usually benign. Isolated skull JXG and other primary bone tumours are difficult to distinguish by imaging examination and clinical symptoms, and histopathology and immunohistochemistry are the most accurate diagnostic methods [6]. JXGs are characterized histopathologically by large amounts of foamy cells and spindle cells with scattered lymphocytes; immunohistochemically, lesional cells express markers of histiocytic dendrocytic differentiation, such as CD68, CD163, and factor XIIIa, with no change observed in CD1a or S100 protein reactivity [6, 7].

## Case presentation

A 15-year-old boy was hospitalized with complaints of unaccountable right migraine for 7 months. There was a mass with tenderness behind his right ear. Before admission, the patient was diagnosed with lymphadenitis based on needle biopsy of the mass in another hospital, and the patient's headache was temporarily relieved with anti-inflammatory treatment. However, the mass gradually increased, his headache continued, and he eventually visited the Neurosurgery Department of our hospital for treatment. The examination revealed a hard swelling of 2 cm with tenderness behind the right ear. No pulse and no sound could be heard through auscultation. The skin on the surface of the swelling was normal without ulceration or redness. Except for the headache and mass behind the right ear, he had no other symptoms. The patient had a normal body temperature, clear mind, and normal muscle tone; pathological reflex was not elicited. No obvious nodules were observed on the skin of other parts of his body. Haematological and biochemical examinations revealed alanine aminotransferase (ALT) of 7 U/L (9–50), aspartate aminotransferase(AST)of 12 U/L (15–40), and basophil percentage (BASO%) of 1.2% (0–1.0); coagulation indicators were in the normal range. The patient had undergone surgery of his left hand due to trauma.

**Fig. 1:**
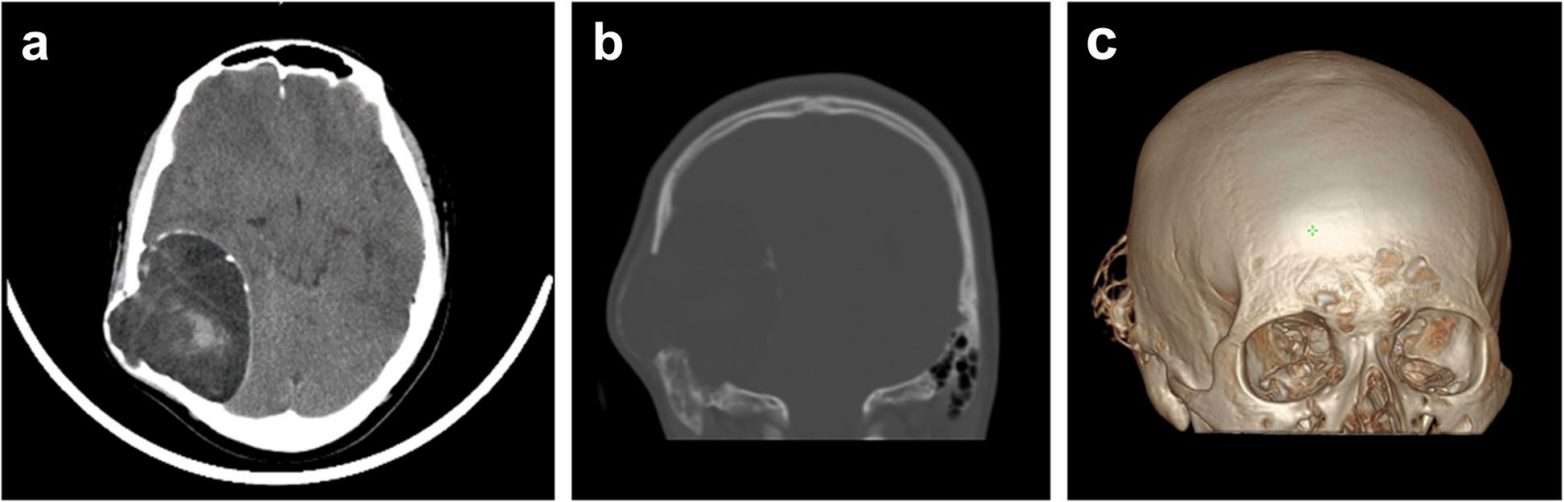
Head computed tomography (CT) (**a**-**c**) scan shows a right temporal mass with cystic and solid components (**a**, axial, soft tissue window). The density of the mass is mostly the same as that of liquid (**a**). Right temporal bone is obviously destroyed (**b**, coronal, bone window) (**c**, three-dimensional reconstruction)

**Fig. 2:**
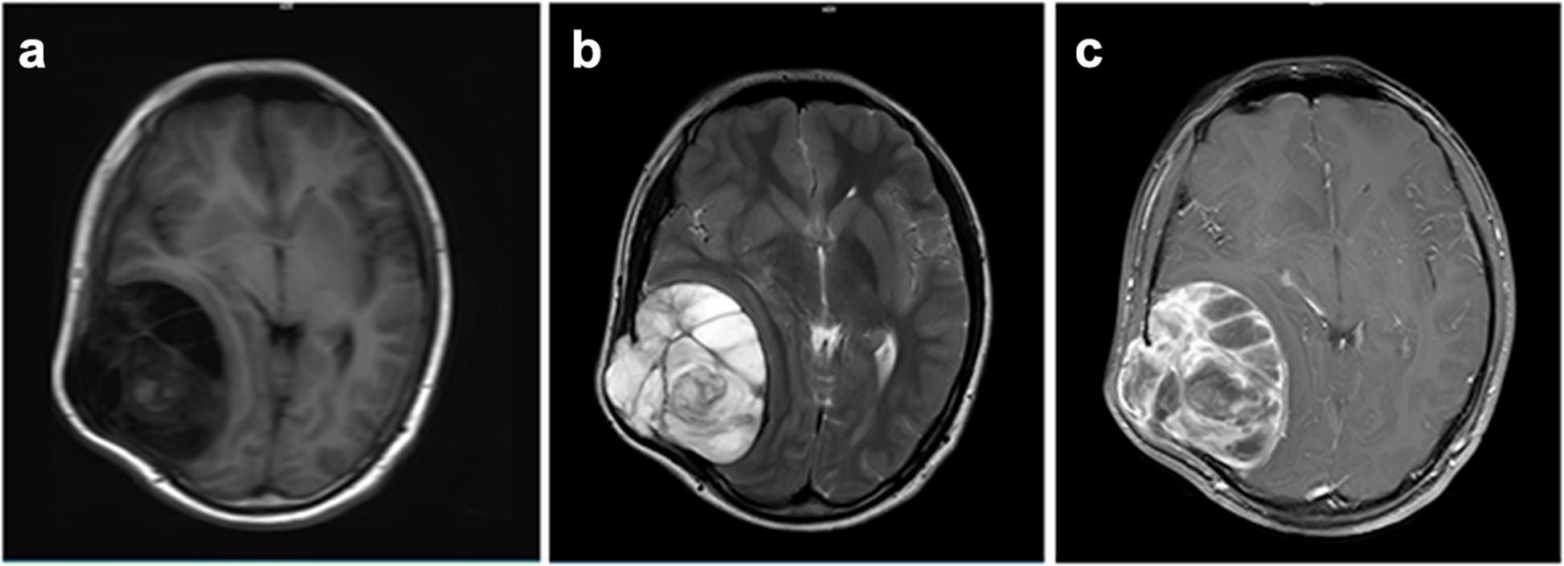
Axial head resonance imaging (MRI) (**a**-**c**) scan shows a right temporal mass with trabecular aspect inside and compressing temporal lobe (**a**-**c**). The mass mainly shows a signal similar to that of liquid on the T1(**a**) and T2 (**b**) weighted images. The mass shows marked contrast enhancement (**c**, T1-weighted fat-suppressed image after contrast enhancement)

A CT scan revealed a cystic solid mass in the right temporo-occipital region (Fig. 1), approximately 7.37 cm × 6.48 cm in size, with smooth margins and calcification. The internal density was uneven, with mainly cystic components. The outer plate of the skull had become thinner and bulged outward. Regarding MRI, the main body of the lesion showed hypointensity on T1-weighted images and hyperintensity on T2-weighted images; the edges were hypointense on T1-weighted images. The edge and trabecular aspect of the lesion showed significant contrast enhancement, though the solid part was uneven and slightly enhanced (Fig. 2). Imaging differential diagnosis included haemangiopericytoma and skull-derived tumour. Further examination was recommended to rule out schwannoma or parasitic infection.

**Fig. 3:**
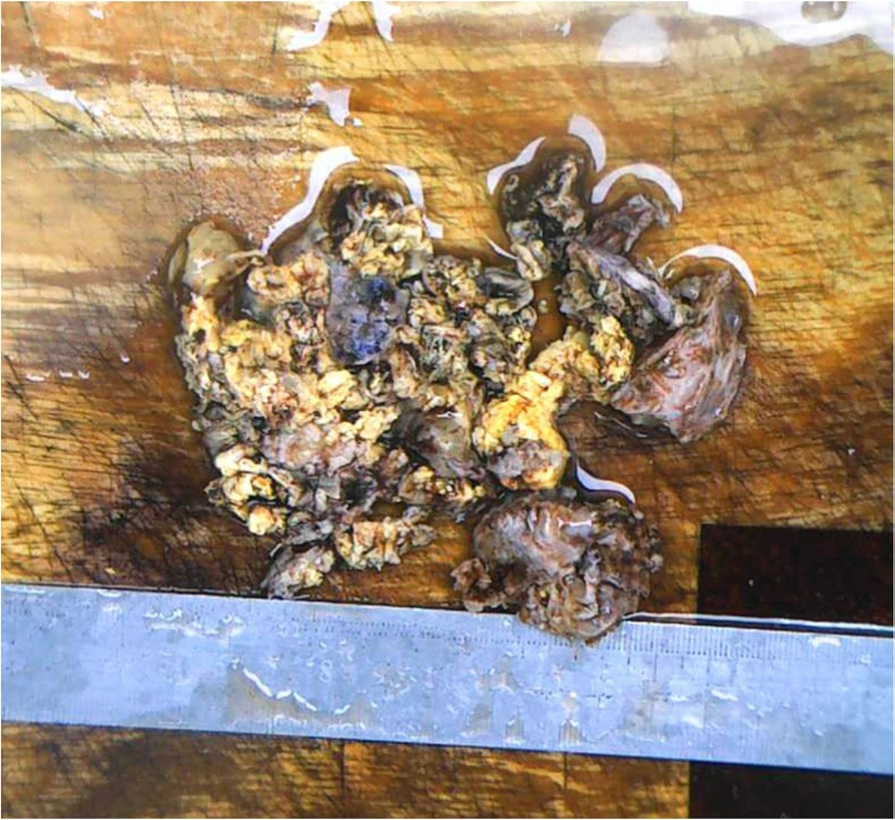
Gross specimen of the tumour

As the imaging picture could not reveal the nature of the mass clearly and the patient had clinical symptoms of headache, he underwent tumour resection and conducted postoperative pathological examination. During the operation, it was found that the tumour was located in the epidural area and originated from the bone at the root of the mastoid. Part of the temporal bone and bone at the root of the mastoid had been destroyed by the tumour, and the tumour had formed on the outside of the skull; it was approximately 8 cm × 8 cm in size and had a flesh-red capsule. The bone around the tumour was small honeycomb-shaped, accompanied by a large amount of oozing blood, and the texture was tough. The blood supply was very rich. The brain tissue was compressed and displaced inward. When the capsule of the tumour was cut, it could be seen that the tumour was cystic and solid. The solid part was grey-yellow, whereas the cystic component was grey-white and gelatinous. The tumour was completely removed (Fig. 3).

**Fig. 4:**
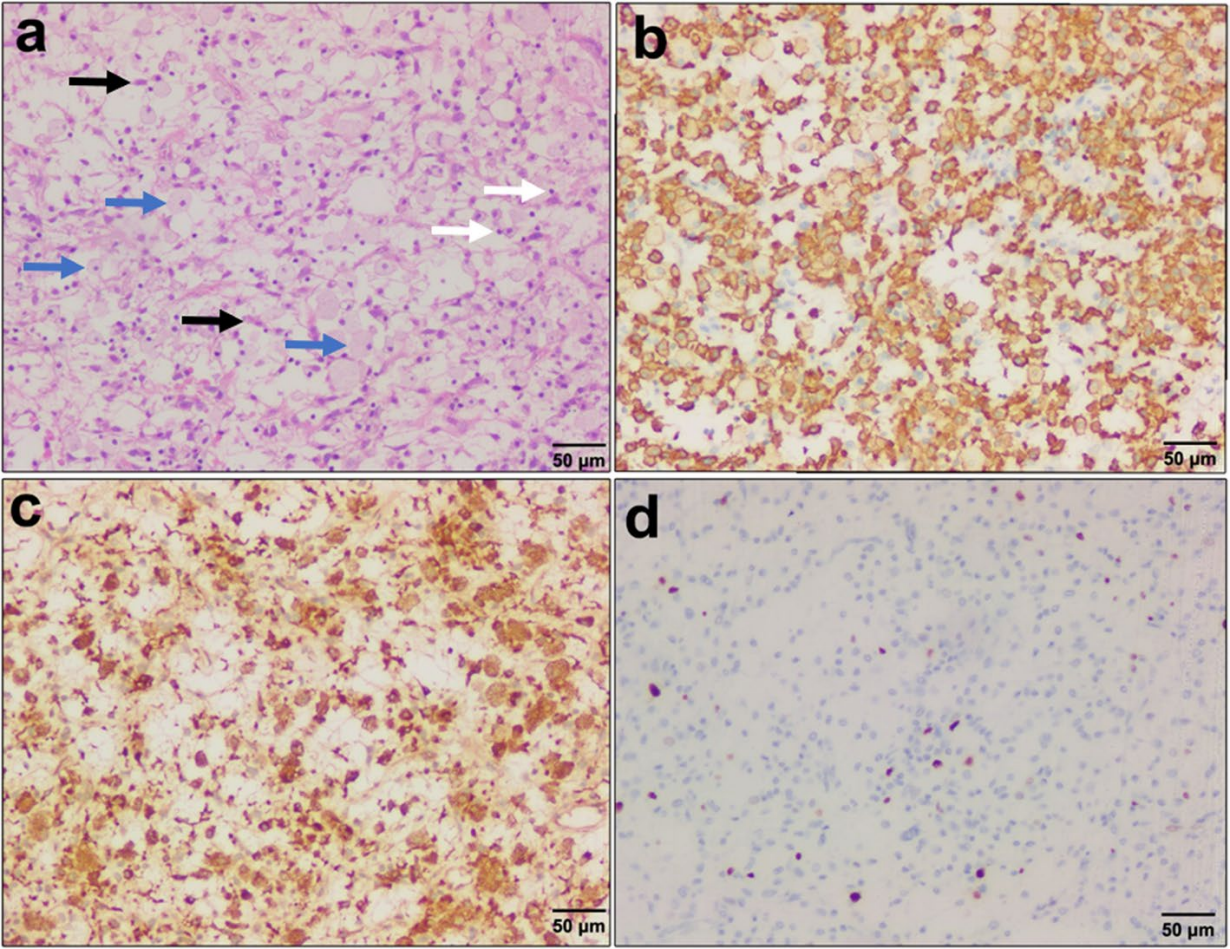
Pathological and immunohistochemical examination of juvenile xanthogranuloma. **a** Under microscopy, short spindle-shaped fibre cells and lymphocyte infiltration as well as foam-like histiocytes were seen (10 × , HE). The black, blue and white arrows indicate spindle cells, foamy cells and lymphocytes, respectively. **b, c, d** Immunohistochemistry showed positive expression of CD163, CD68 and Ki-67 in cells

Under microscopy, a large number of histiocytes, foam-like and containing lipids, were surrounded by fibrocytes and lymphocytes. In the background of myxoedema, the fibrocytes were short-spindle-shaped; nuclear divisions were rare (Fig. 4a). Immunohistochemical results were as follows: CD163 ( +) (Fig. 4b), CD68 ( +) (Fig. 4c), CD3 (scattered +), CD20 (scattered +), Ki-67 (approximately 3% +) (Fig. 4d), CDla (-), S-100 (-), CD207 (Langerin) (-), GFAP (-), PCK[AE1/AE3] (-), EMA ( +), p40 (-), P63 (-), Olig-2 (-). The diagnosis was xanthogranuloma.

The patient did not receive any chemotherapy or radiotherapy, and the postoperative prognosis was good, without obvious complications. Skull repair surgery was performed nine months later. During the 19-month follow-up period, the patient had no obvious headache and showed no obvious signs of recurrence on imaging examination.

## Discussion and conclusions

JXG usually manifests as skin lesions, which can regress spontaneously without any treatment. Although most JXGs are benign, death may occur with systemic JXG involving multiple internal organs [8]. Besides the cutaneous and systemic JXG presentation, JXG can only involve solitary extracutaneous presentation. Solitary osseous lesions are rare. To the best of our knowledge, only 4 other cases of solitary skull JXG have been reported (Table 1).

**Table 1:** Solitary Juvenile Xanthogranuloma (JXG) with Skull-Based Localization

Author	Year	Site	Age	Sex	Treatment	Response	Outcome
Puerta et al. [3]	2013	Temporal bone	3 m	M	Total surgical removal	CR	NED 3 y
De Paula et al. [1]	2010	Occipital bone	18 m	F	Partial surgical removal, Chemotherapy, RT	PR	SD 3 y
Cornips et al. [4]	2009	Temporal muscle and bone	2 m	M	Partial surgical removal	CR	SD 21 m
Farrugia et al. [5]	1997	Temporal bone	2 y	F	Partial surgical removal	CR	SD 6 m
Present case	2021	Temporal bone	15 y	M	Total surgical removal	CR	NED 19 m

*M* Male, *F* Female, *y* Year(s), *m* Month(s), *RT* Radiotherapy, *CR* Complete response, *PR* Partial response, *NED* No evidence of disease, *SD* Stable disease

This disease should be differentiated from LCH (Langerhans cell histiocytosis), which is also a histiocytosis. As a form of non-Langerhans cell histiocytosis, JXG has an indolent clinical process, whereas LCH is more aggressive and has worse prognosis. Overall, JXG imaging performance lacks specificity. Immunohistochemistry helps in distinguishing JXG from LCH. XIIIa, CD68, CD163, fascin and CD14 positivity and CD1a, S100, Langerin and/or Birbeck particle negativity may help in diagnosing JXG [9]. LCH cells are positive for CD1a and S100 but negative for XIIIa. JXG of the temporal bone needs to be differentiated from some other relatively common lesions (such as epidermoid cyst, osteoblastoma, and chondroblastoma), which usually depends on histopathology and immunohistochemistry. Pathological examination may reveal a large number of foamy cells, spindle cells and CD68 + , CD1a − , and S100 − results.

Treatment of solitary skull JXG includes resection, chemotherapy or radiotherapy. Total resection usually has a favourable outcome. As shown in Table 1, both patients who underwent total surgical removal had no evidence of the disease during 19–36 months of follow-up [3]. In contrast, two of the three patients with partial resection were in stable condition during 6–21 months of follow-up [4, 5]. In only one case was recurrence noted after major resection, and the condition became stable through chemotherapy, reoperation, and radiotherapy [1]. Because isolated skull JXG is extremely rare, other isolated intracranial or vertebral JXG treatment methods can be considered. Among isolated intracranial or vertebral JXGs, most patients undergoing complete resection had a good prognosis, though a small portion with partial resection had local recurrence [1]. Among those with relapse, the patients generally remained in a stable condition after radiotherapy and chemotherapy. Therefore, for isolated skull JXG, total surgical resection is the optimal treatment. If total resection is difficult to achieve, chemotherapy should be considered as a supplementary treatment. Radiotherapy is an option for chemotherapy-refractory cases.

In summary, isolated skull JXG is rare. However, when there is an intracranial space-occupying lesion accompanied by skull damage, especially when the patient is an infant, child or adolescent under the age of 20, this disease should be included in the scope of differential diagnosis. Complete resections usually have excellent outcomes. If total resection is difficult to achieve, partial resection combined with chemotherapy or radiotherapy is an effective treatment. The patient in the present case underwent total resection and has exhibited no sign of recurrence thus far. We will continue to follow up and observe this patient.

## Data Availability

The datasets used and/or analyzed during the current study are available from the corresponding author on reasonable request.

## Abbreviations

JXG: Juvenile xanthogranuloma
CT: Computed tomography
MRI: Magnetic resonance Imaging
ALT: Alanine aminotransferase
AST: Aspartate aminotransferase
BASO: Basophils
CNS: Central nervous system
M: Male
F: Female
y: Year(s)
m: Month(s)
RT: Radiotherapy
CR: complete response
PR: Partial response
NED: No evidence of disease
SD: Stable disease
